# Long noncoding RNAs in vascular smooth muscle cells regulate vascular calcification

**DOI:** 10.1038/s41598-019-42283-x

**Published:** 2019-04-10

**Authors:** Geon Jeong, Duk-Hwa Kwon, Sera Shin, Nakwon Choe, Juhee Ryu, Yeong-Hwan Lim, Jaetaek Kim, Woo Jin Park, Hyun Kook, Young-Kook Kim

**Affiliations:** 1 Basic Research Laboratory for Cardiac Remodeling Research Laboratory, Chonnam National University Medical School, Jeollanam-do, Republic of Korea; 2 Department of Biochemistry, Chonnam National University Medical School, Hwasun, Jeollanam-do 58128 Republic of Korea; 3 Center for Creative Biomedical Scientists, Chonnam National University Medical School, Hwasun, Jeollanam-do 58128 Republic of Korea; 4 Department of Pharmacology, Chonnam National University Medical School, Hwasun, Jeollanam-do 58128 Republic of Korea; 5 Division of Endocrinology and Metabolism, Department of Internal Medicine, College of Medicine, Chung-Ang University, Seoul, Republic of Korea; 6 College of Life Sciences, Gwangju Institute of Science and Technology (GIST), Gwangju, Republic of Korea

## Abstract

Vascular calcification is characterized by the accumulation of hydroxyapatite crystals, which is a result of aberrant mineral metabolism. Although many clinical studies have reported its adverse effects on cardiovascular morbidity, the molecular mechanism of vascular calcification, especially the involvement of long noncoding RNAs (lncRNAs), is not yet reported. From the transcriptomic analysis, we discovered hundreds of lncRNAs differentially expressed in rat vascular smooth muscle cells (VSMCs) treated with inorganic phosphate, which mimics vascular calcification. We focused on Lrrc75a-as1 and elucidated its transcript structure and confirmed its cytoplasmic localization. Our results showed that calcium deposition was elevated after knockdown of Lrrc75a-as1, while its overexpression inhibited calcium accumulation in A10 cells. In addition, Lrrc75a-as1 attenuated VSMCs calcification by decreasing the expression of osteoblast-related factors. These findings suggest that Lrrc75a-as1 acts as a negative regulator of vascular calcification, and may serve as a possible therapeutic target in vascular calcification.

## Introduction

Vascular calcification is caused by an imbalance of mineral metabolism, especially calcium phosphate metabolism^1^. It decreases vessel wall tension and increases vascular stiffness, thereby increasing the risk of myocardial ischemia, heart failure, arrhythmias, and other cardiovascular diseases^2^. Vascular calcification includes several major types including medial arterial calcification, intimal atherosclerosis, and arterial calcification of chronic kidney diseases^3^. Medial arterial calcification is characterized by extensive calcium precipitation in the arterial tunica media, which increases vessel stiffness. Intimal atherosclerosis is initiated by production of eccentric atherosclerosis plaque, followed by hyperplasia of the intimal smooth muscle cells that leads to reduction of the lumen area. Arterial calcification of chronic kidney diseases exhibits features of both medial calcification and intimal atherosclerosis. Commonly, vascular calcification results in loss of vessel elasticity and negatively affects cardiovascular hemodynamics^3,4^. Accordingly, a detailed understanding of the regulatory mechanism of vascular calcification is crucial for the treatment of diverse cardiovascular diseases.

Previously, vascular calcification was regarded as a passive consequence of degenerative diseases, but recent studies have demonstrated that as an active process, vascular calcification shares several features of bone formation^5^. Among the osteoblast-related factors, Runt-related transcription factor 2 (Runx2), bone morphogenetic proteins (BMPs), and msh homeobox 2 (Msx2) have been shown to be upregulated in calcified vessels. On the contrary, contractile markers, such as smooth muscle 22 alpha and alpha smooth muscle actin, which retain the myofilament structure, are downregulated in calcified vessels^6^. This indicates that mature vascular smooth muscle cells (VSMCs) can alter their phenotype from a contractile to an osteoblastic/chondrogenic phenotype, a phenomenon called as ‘phenotype switching’^7^.

The importance of noncoding RNA was emphasized in the Encyclopedia of DNA Elements (ENCODE) project, where it was revealed that the majority of the human genome consists of noncoding regions; however, diverse transcripts are produced from this region^8,9^. Recent studies have demonstrated that a variety of noncoding RNAs are involved in developmental processes, cellular physiology, and in the progression of various diseases^10–12^. Noncoding RNAs are classified into small or long noncoding RNAs (lncRNAs) depending on their nucleotide (nt) length. LncRNAs are longer than 200 nt and share many features of mRNAs, except for their protein-coding potential. Most lncRNAs are transcribed by RNA polymerase II, capped, and spliced, and some of them are polyadenylated^11,13^. The functions of lncRNAs are poorly understood compared to those of small noncoding RNAs due to their diverse and complex working mechanism^14^. The main working mechanism of lncRNAs is through interaction with diverse proteins and microRNAs (miRNAs). Especially, it has been suggested that lncRNAs are efficient regulators of protein functions^11,15^. One protein consisting of 100 amino acids was predicted to capture one or two proteins, while a 100 nt-long RNA would trap around five to ten proteins at the same time, suggesting that lncRNAs are efficient regulator of proteins. Moreover, in the cytoplasm, lncRNAs can capture many miRNAs at the same time due to their long sequences. Since a number of lncRNAs have been found that are comparable to a number of mRNA genes, it can be expected that lncRNAs may have important roles in diverse cellular processes and many diseases.

While many studies have identified the function of miRNAs in cardiovascular diseases, the role of lncRNAs in cardiovascular diseases, including vascular calcification is less understood^16,17^. Until now, there is no lncRNA that has been reported to be involved in vascular calcification. It was reported that smooth muscle and endothelial cell enriched migration/differentiation-associated lncRNA (SENCR) and myocardin-induced smooth muscle lncRNA, inducer of differentiation (MYOSLID) lncRNAs control the phenotypic switching of VSMCs to maintain their contractile phenotype^18,19^. Another lncRNA, taurine up-regulated gene 1, was shown to upregulate the expression of Runx2 through sponging miR-204-5p, and increase osteoblast differentiation of aortic valve interstitial cells^20^. However, the lncRNA that is directly involved in the regulation of vascular calcification is not yet identified.

In this study, we identified diverse lncRNAs that are differentially expressed in VSMCs during vascular calcification through RNA sequencing. Subsequent functional studies showed that among the selected lncRNAs, Lrrc75a-as1 regulated calcium deposition in VSMCs. Hence, our study revealed that Lrrc75a-as1 can be considered as a possible therapeutic target for the treatment of diverse diseases related with vascular calcification.

## Results

### Identification of candidate lncRNAs involved in vascular calcification

**Figure 1:**
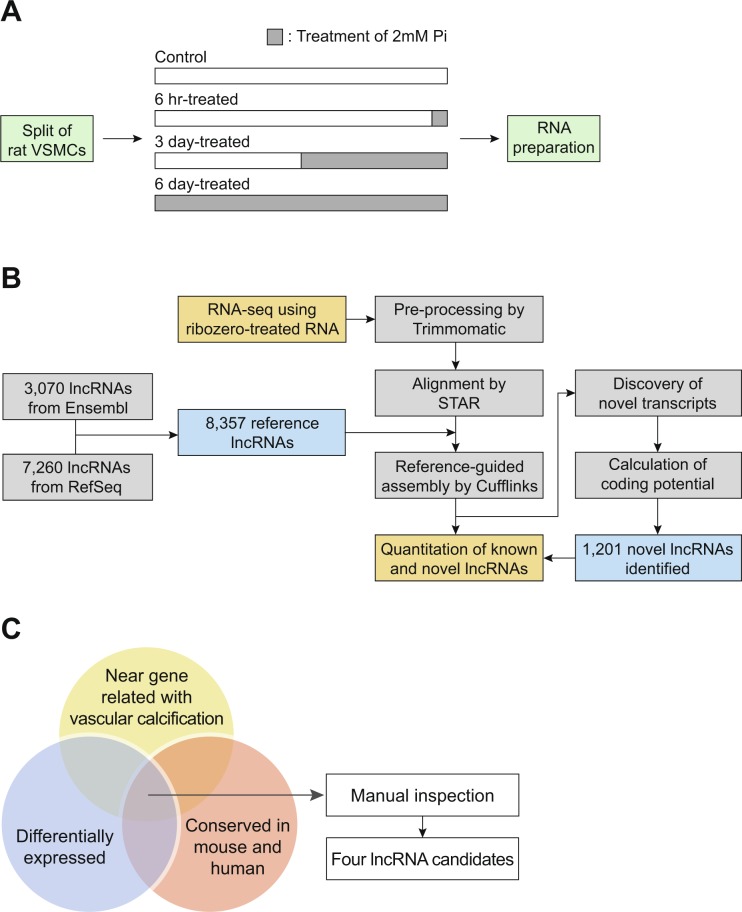
Identification of lncRNAs involved in vascular calcification. (**A**) Experiment scheme. After splitting the primary-cultured rat VSMCs into four dishes, they were treated with 2 mM Pi for different time periods. Time point of Pi treatment was adjusted such that all cells had the same growth period. (**B**) Schematic representation of lncRNA analysis. RNA sequencing and its following analyses steps are illustrated. Refer to the Materials and methods section for details. (**C**) Three criteria to select the candidate lncRNAs: differential expression of lncRNAs among the samples, conservation among species, and existence of near gene in genomic context with known function related with vascular calcification. Refer to the main text for details.

To identify the lncRNAs involved in vascular calcification, we utilized primary-cultured VSMCs. VSMCs were treated with 2 mM Pi for different time periods (Fig. 1A). Treatment of the cells with high concentration of Pi efficiently recapitulates vascular calcification, as shown in our previous study^21^. We extracted total RNA from the samples, depleted the ribosomal RNAs, and performed total RNA sequencing (Fig. 1B). The pathway analysis for the decreased mRNAs after Pi treatment showed that the genes involved in the VSMC contractions were most highly decreased^22,23^, suggesting that the phenotype of VSMCs was successfully changed in our experimental condition (Supplementary Fig. 1). There is no representative annotation for rat lncRNAs. Therefore, we collected the annotations from two public databases, Ensembl and RefSeq, for quantification of the expression level of lncRNAs^24,25^. These annotations were merged, which resulted in a new reference annotation with 8,357 lncRNA genes (Fig. 1B). Using this annotation, we analyzed the RNA sequencing data, and discovered 1,201 novel lncRNA genes expressed in rat VSMCs. We quantified the expression level of known and novel lncRNAs in our samples, and calculated their expression change after induction of vascular calcification (Supplementary Table 1).

We used the following three criteria for the selection of candidate lncRNAs for further studies: (1) differential expression of lncRNAs in Pi-treated samples compared to that in untreated control samples, (2) conservation of the locus of lncRNA genes in the genome across rat, mouse, and human with respect to nearby protein-coding genes, and (3) the existence of a previous study for the neighboring genes, wherein their function in calcification, calcium regulation, or related processes has been reported (Fig. 1C). We included this third criterion because there is a higher tendency for the closely located genes to be regulated by common regulatory factors. These criteria resulted in the selection of four lncRNA candidates. Because the gene structure of lncRNAs in rat is poorly annotated, we first used the most similar expressed sequence tag (EST) as the name of each candidate lncRNA, which include FM045041, BG663343, BQ204485, and CR471446.

We examined the genomic information of these four candidate lncRNAs in human. Our candidates, FM045041, BG663343, BQ204485, and CR471446 were already named as Snhg1, Linc00116, Snhg16, and Lrrc75a-as1 in human, respectively. Therefore, we used these annotated names in the following description. Snhg1 is located adjacent to Slc3a2, which produces the heavy chain of CD98 (CD98hc). CD98hc is connected with integrin, which influences VSMC proliferation and survival^26^. St6galnac2, a neighboring gene of Snhg16, mediates the transfer of sialic acid from CMP-Neu5Ac to O-linked oligosaccharides of fetuin, which is a known inhibitor of vascular calcification^27,28^. Linc00116 was detected near nephrocystin 1 (Nphp1). Nphp1 is involved in the regulation of renal phosphate excretion^29^. Increased serum phosphate levels tend to accelerate vascular calcification in patients with chronic renal failure^30^. Finally, Lrrc75a-as1 has Trpv2 as its neighboring gene in the genomic context. Trpv2 is an important membrane protein that forms the non-selective cation channel, which is stimulated by hypotonic solution-induced cell swelling in VSMCs, and ascends calcium influx in VSMCs^31^.

**Figure 2:**
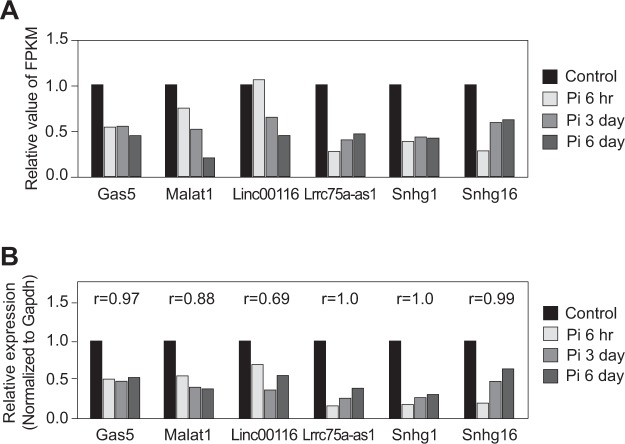
Confirmation of the expression change of lncRNAs. (**A**) Fold change of lncRNA expression from RNA sequencing data. The average FPKM values from duplicate samples were obtained and fold changes compared to control were calculated. (**B**) Validation of the expression level of lncRNAs by qRT-PCR. The correlation coefficients r between relative expression levels measured by RNA sequencing and by qRT-PCR were calculated.

We next validated the expression level of the four candidate lncRNAs by qRT-PCR, and compared these levels to those from RNA sequencing data (Fig. 2). Growth arrest-specific 5 (Gas5) and metastasis associated lung adenocarcinoma transcript 1 (Malat1), which were previously shown to have functional roles in vascular smooth muscles, were selected as control lncRNAs^32,33^. The expression level of all lncRNA candidates was consistent with RNA sequencing results. Therefore, we focused on these four candidate lncRNAs for the following experiments.

### Analysis of gene structure and localization of candidate lncRNAs

**Figure 3:**
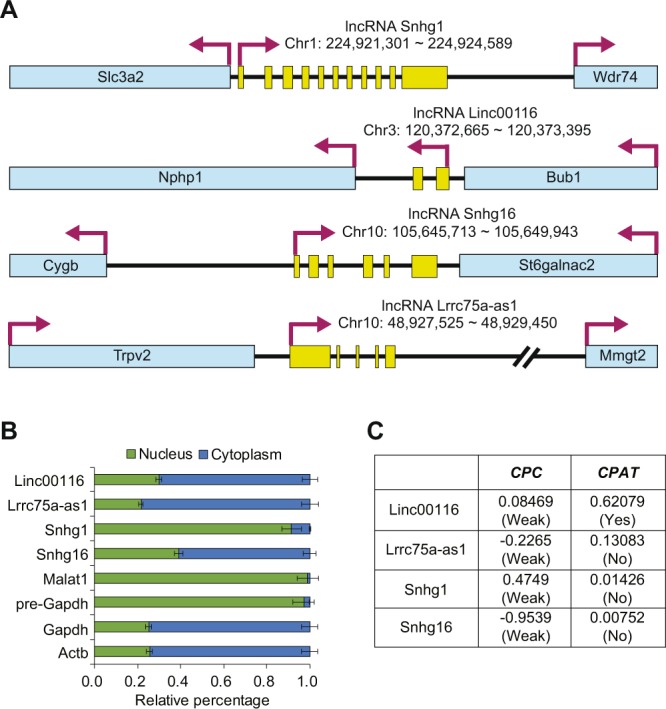
Characterization of candidate lncRNAs. (**A**) Genomic information of selected lncRNAs. By performing 5′ and 3′ RACE experiments and subsequent sequencing, the transcription start and end positions, and splice junctions were identified. The nucleotide position is based on rn6 (July 2014) genome assembly. Yellow boxes represent the exons of lncRNAs. Blue boxes represent the locus of nearby protein-coding genes. (**B**) The subcellular distribution of lncRNAs. LncRNA levels were measured in the nuclear and cytoplasmic fractions of A10 cells. Results showed that Linc00116, Lrrc75a-as1, and Snhg16 were mainly localized in the cytoplasm. Snhg1 was localized in the nucleus. Malat1 and pre-Gapdh were used as nuclear controls, while mature Gapdh and Actb were used as cytoplasmic markers. The error bars indicate standard error of triplicate measurements (*n* = 3). (**C**) Protein-coding potential of lncRNAs. The protein-coding potential of candidate lncRNAs was evaluated by CPC^36^ and CPAT^37^.

The sequences of lncRNAs are poorly defined, especially in rat. Therefore, we performed 5′ and 3′ RACE experiments to identify the full-length sequences and transcript structures of the selected lncRNAs (Fig. 3A and Supplementary Table 2). From the analysis, we obtained the transcript structure of full-length lncRNAs, including the transcription start site, termination site, and splicing sites.

The localization of lncRNAs in cells give us an indication of their possible working mechanism^34^. Therefore, we analyzed the subcellular localization of lncRNAs in A10 cells, a cell line derived from vascular smooth muscle, through cell fractionation and transcript measurement. We included control marker transcripts, which were known to reside in the nucleus or cytoplasm exclusively. For the nucleus-residing transcripts, Malat1^35^ and pre-Gapdh were selected. Conversely, mature Gapdh and Actb were selected as the representative cytoplasm transcripts. Our data demonstrated that Linc00116, Lrrc75a-as1, and Snhg16 predominantly resided in the cytoplasm, while Snhg1 resided in the nucleus (Fig. 3B).

To identify the candidate lncRNA with a possible coding sequence, we assessed their protein-coding potential using Coding Potential Calculator (CPC)^36^ and Coding Potential Assessment Tool (CPAT)^37^. Lrrc75a-as1, Snhg1, and Snhg16 showed low coding potential in both bioinformatics tools (Fig. 3C). Because a moderate coding potential was observed for Linc00116 from CPAT algorithm, we excluded this lncRNA from all further analyses.

### Lrrc75a-as1 reduces calcium deposition in the cells

**Figure 4:**
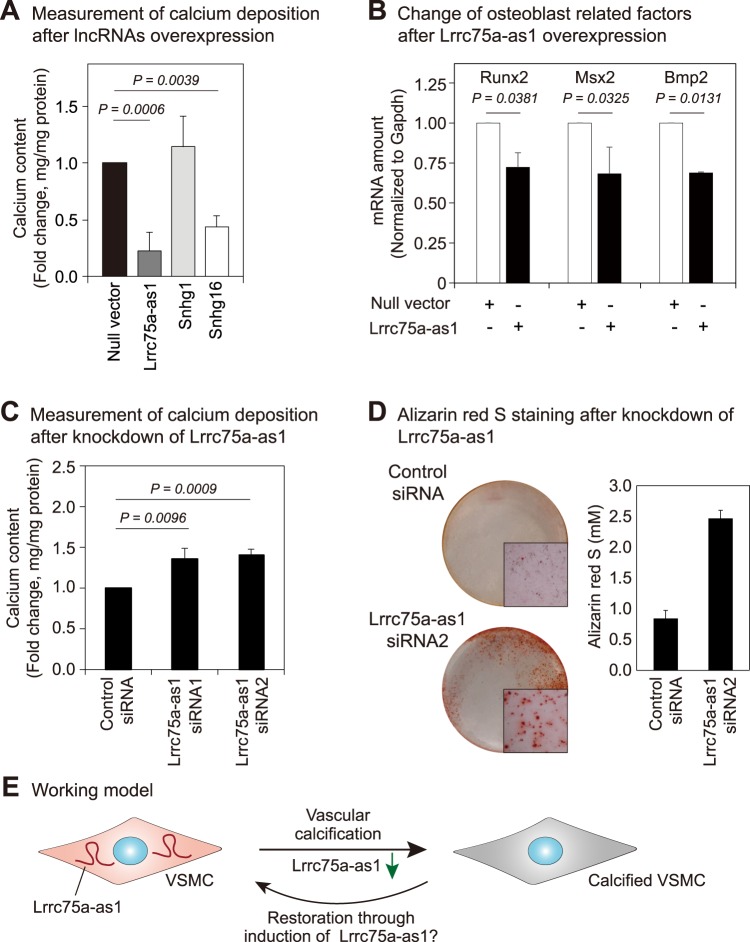
Lrrc75a-as1 inhibits vascular calcification. (**A**) Measurement of calcium deposition after overexpression of candidate lncRNAs. Compared to other lncRNAs, overexpression of Lrrc75a-as1 most significantly changed the calcium content. Error bars indicate standard error between four independent experiments with triplicates in each experiment. P value was calculated by a two-sided paired *t*-test. (**B**) Expression change of the osteoblast-related factors after Lrrc75a-as1 overexpression. The expression of osteoblast-related factors, including Runx2, Msx2, and Bmp2 was measured. Error bars indicate standard error between three independent experiments. (**C**) Measurement of calcium deposition after knockdown of Lrrc75a-as1. Six independent experiments were performed, and the P value was calculated by a two-sided paired *t*-test. (**D**) Determination of calcification with Alizarin red S staining. After the knockdown of Lrrc75a-as1, the calcium deposits were measured with Alizarin red S dye. The pictures of cell culture dishes and microscopic images (50×) were shown. The amounts of Alizarin red S-stained mineralization were quantified from three cell culture dishes and error bars indicate the standard errors. (**E**) Working model. The expression level of Lrrc75a-as1 is reduced during vascular calcification in VSMCs. Upregulation of Lrrc75a-as1 expression may switch the VSMC phenotype from an osteoblastic/chondrogenic to a contractile phenotype, and mitigate calcium accumulation.

To find the lncRNAs involved in vascular calcification, we cloned the sequences of three selected lncRNAs into a plasmid based on the full-length sequence identified from RACE experiments (Fig. 3A). Since the expression of three candidate lncRNAs was decreased after Pi treatment (Fig. 2), we hypothesized that overexpression of these lncRNAs decreases the accumulation of calcium in cells. We compared the calcium content in A10 cells under calcification medium between the control and lncRNA-overexpressing samples. Our results demonstrated that the lncRNAs, Lrrc75a-as1 and Snhg16, diminished calcium deposition in A10 cells (Fig. 4A). Because Lrrc75a-as1 reduced the calcium content more than Snhg16, we selected this lncRNA for further studies. We also found that overexpression of Lrrc75a-as1 markedly reduced the mRNA expression levels of the osteoblast-related factors, Runx2, Msx2, and Bmp2. This result suggests that Lrrc75a-as1 is involved in the regulation of VSMC calcification by decreasing the expression of osteoblast-related factors (Fig. 4B).

To reinforce the possible role of Lrrc75a-as1 in vascular calcification, we also performed an RNA interference experiment to deplete Lrrc75a-as1. We designed two different siRNAs targeting different regions of Lrrc75a-as1 and confirmed efficient depletion of Lrrc75a-as1 by both siRNAs (Supplementary Fig. 2). We found that knockdown of Lrrc75a-as1 augmented calcium deposition in A10 cells (Fig. 4C). Moreover, staining of the cells with Alizarin red S showed that the calcium deposits increased when the Lrrc75a-as1 was depleted (Fig. 4D). Taken together, our results suggest that Lrrc75a-as1 inhibits vascular calcification through regulation of VSMC phenotype switching (Fig. 4E).

The loci of human and mouse orthologs of Lrrc75a-as1 contain the small nucleolar RNA (snoRNA) sequences for Snord49a, Snord49b, and Snord65 (Supplementary Fig. 3). Therefore, it is plausible that Lrrc75a-as1 might be a host gene to produce these snoRNAs, which might play a role in vascular calcification. Until now, there are no functional studies for these snoRNAs; hence, future studies are needed to investigate their role in vascular calcification.

### Analysis of the regulatory network of Lrrc75-as1

**Figure 5:**
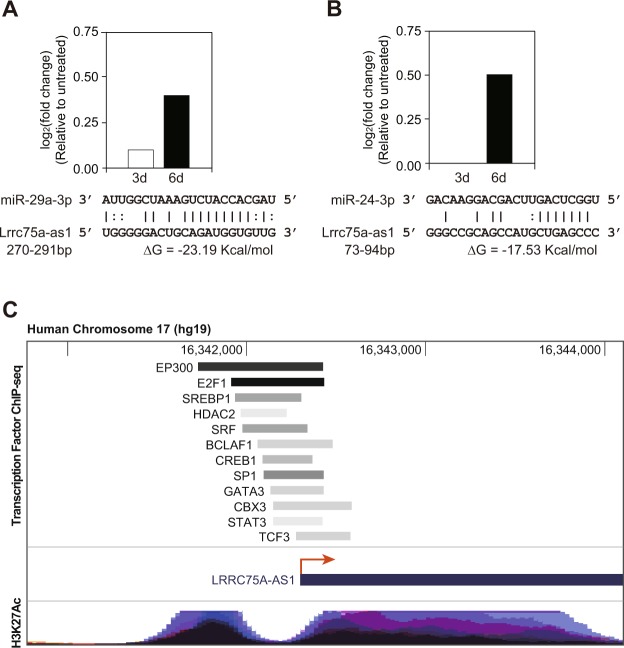
Analysis of the regulatory network of Lrrc75a-as1. (**A**,**B**) Expression change of (**A**) miR-29a-3p and (**B**) miR-24-3p during vascular calcification, and their sequences predicted to bind to that of Lrrc75a-as1 are shown. The expression change of miRNAs between untreated and Pi-treated samples for three or six days was calculated. The binding position was predicted from miRNA_Targets server^38^. (**C**) Analysis of transcription factors in the promoter of human Lrrc75a-as1. The position of ChIP-seq signals for each transcription factor and H3K27Ac was obtained using the UCSC Genome Browser (http://genome.ucsc.edu)^58^. The histone marker H3K27Ac is usually associated with active enhancers^59^.

Cytoplasmic lncRNA generally acts as a miRNA sponge. Therefore, we hypothesized the possible working mechanism of Lrrc75a-as1 as a miRNA regulator. We analyzed the putative target miRNAs for Lrrc75a-as1 using the miRNA target prediction tool, miRNA_targets, which analyzes thermodynamic stability and complementarity between noncoding RNA and miRNAs^38^. From this analysis, 47 miRNAs were discovered to interact with Lrrc75a-as1 in a sequence-specific manner. To select the miRNAs that show an inverse correlation profile with Lrrc75a-as1, we utilized the expression profiles of miRNAs during the calcification of rat VSMCs (manuscript in preparation). Moreover, we only selected miRNAs that have been reported in previous studies involving vascular calcification or other related functions. Based on this, we selected two miRNAs, miR-29a-3p and miR-24-3p, which have binding sites on Lrrc75a-as1 (Fig. 5A,B). Among these target miRNAs of Lrrc75a-as1, miR-29a-3p has been identified to regulate VSMC calcification by targeting ADAMTS-7^39^, while the miR-24 family members have been reported to have significant function in cardiovascular diseases^40^. Especially, miR-24-3p regulates the phenotype transition of VSMCs from a synthetic to a contractile phenotype by targeting Tribbles homolog 3^41^. Thus, increased activity of miR-24-3p resulting from a decrease in Lrrc75a-as1 level may facilitate vascular calcification. We thereby propose that Lrrc75a-as1 may regulate vascular calcification through interaction with these miRNAs.

Because the level of Lrrc75a-as1 transcripts decreases during vascular calcification, we expected that any transcription factor related with vascular calcification might bind to the promoter of Lrrc75a-as1 and regulate its transcription. We utilized the chromatin immunoprecipitation followed by sequencing (ChIP-seq) data published in the ENCODE project^9^. Because the ChIP-seq data was mainly obtained from human cells, we analyzed the promoter of human homolog of Lrrc75a-as1 (Supplementary Fig. 3). Among the various transcription factors, we only selected the transcription factors whose regulatory roles in VSMCs have been reported. We found that many factors related with VSMCs directly bind to the promoter of Lrrc75a-as1 (Fig. 5C). Strikingly, these transcription factors, including serum response factor (SRF), cAMP responsive element binding protein 1 (CREB1), signal transducer and activator of transcription 3 (STAT3) have been previously shown to be associated with vascular calcification^42–44^. Thus, our findings revealed that during vascular calcification, the amount or activity of these transcription factors changes, and this in turn decreases the expression of Lrrc75a-as1.

## Discussion

In this study, we observed that the expression levels of numerous lncRNAs were changed during vascular calcification, as evidenced by the RNA sequencing data. Compared to human or mouse, the sequence and structure of rat lncRNAs are less identified. Therefore, we merged two most highly used annotations for rat genes. In RefSeq and Ensembl annotations, there were 7,260 and 3,070 annotated lncRNA genes, respectively (Fig. 1B). Moreover, the integration of annotations from these two databases resulted in the annotation for 8,357 lncRNA genes, suggesting that the annotations of many lncRNAs only exist in one database exclusively. The huge difference in lncRNA numbers between the two major databases, and less overlap of annotations between them suggests that the annotation of lncRNAs in organisms other than human and mouse is poorly established. As a result, from our rat VSMCs samples, we identified 1,201 novel lncRNAs, which accounts for one-seventh of the reference database that we used for analysis (Supplementary Table 1). This suggests that for the analysis of lncRNAs in organisms other than human or mouse, there are many lncRNAs yet to be identified, and RNA sequencing rather than microarray is a more suitable method to discover novel lncRNA candidates.

From the RNA sequencing data, we selected four candidate lncRNAs whose expression levels were decreased during vascular calcification. Among these candidates, changes in the expression level of Lrrc75a-as1 influenced calcium deposition in VSMCs. In previous studies, Lrrc75a-as1 was shown to be expressed in normal villous maturation, human osteosarcoma, breast cancer, and gastric cancer tissues^45–48^. Lrrc75a-as1 was also predicted as a prognostic factor in acute myeloid leukemia based on statistical analysis^49^. However, an experimental study of the role of Lrrc75a-as1 in VSMCs has not yet been reported. In this study, we found that Lrrc75a-as1 suppresses calcium accumulation in VSMCs, suggesting that it functions as an inhibitor of vascular calcification. For Snhg16 lncRNA whose overexpression decreased calcium deposition in the cells (Fig. 4A), we also observed a slight increase of the calcium contents when it was depleted in the cells (Supplementary Figs 2 and 4a). However, the marker genes related to osteoblast differentiation did not change significantly after the overexpression of Snhg16 (Supplementary Fig. 4b). Thus, this lncRNA might regulate the calcium content in the cells with a different mechanism of Lrrc75a-as1.

At this point, the working mechanism about how Lrrc75a-as1 regulated the calcium metabolism is unknown. To surmise a possible regulatory target of Lrrc75-as1, we analyzed the subcellular localization of this lncRNA. Our data of subcellular fractionation suggests that Lrrc75a-as1 mainly acts in the cytoplasm (Fig. 3B). Previous studies suggested that the lncRNAs in the cytoplasm mainly exert their function through suppressing miRNAs. Interestingly, many miRNAs were predicted to bind the Lrrc75a-as1 sequence. Among these, there were miR-29a-3p and miR-24-3p miRNAs, which were reported to have important functions in the physiology of VSMCs (Fig. 5). Further investigation about the target genes modulated by these miRNAs in VSMCs is required to identify the detailed working mechanism of Lrrc75a-as1 in vascular calcification.

The analysis of upstream signaling may give us a hint about the function of lncRNAs. Therefore, we analyzed the promoter region of Lrrc75a-as1 lncRNA. Because this lncRNA is evolutionarily conserved among rat, mouse, and human, we investigated the transcription factors reported to bind at the promoter region of human Lrrc75a-as1 (Fig. 5C). This analysis led us to identify the transcription factors such as SRF, CREB1, and STAT3 as possible factors which may regulate the transcription of Lrrc75a-as1. It was reported that many target genes of SRF were related to calcium homeostasis^42^. Moreover, the knockdown of CREB1 attenuated low potassium-induced VSMC calcification^43^. It was also shown that suppression of STAT3 inhibited accelerated calcification by interleukin-6^44^. Because of the strong association of these factors to calcification, experimental validation of its regulation on Lrrc75a-as1 will be an important subject to elucidate the regulatory network involving lncRNAs during vascular calcification.

We also examined the expression level of Trpv2, a neighboring gene of Lrrc75a-as1. The marginal increase of Trpv2 mRNA was observed after induction of vascular calcification as analyzed from our RNA-seq data (Supplementary Fig. 5). It is probable that small amounts of Lrrp75-as1 transcript in the nucleus might be involved in the regulation of Trpv2.

A previous study reported Bmp2, Msx2, and Runx2 as osteoblast-related factors in calcified vessels^50^. Bmp2 is a crucial factor in bone formation and vascular calcification. Moreover, Bmp2 facilitates osteoblast differentiation, bone mineralization, atherosclerosis, and vascular calcification. Bmp2 begins calcification signaling in VSMCs and subsequently conducts transactivation of Msx2 and Runx2 in VSMCs^51^. Msx2 plays an important role in intramembranous bone formation, which is similar to the calcification of tunica media. Its role in vascular calcification has also been demonstrated^52^. Runx2 has been shown to regulate vascular calcification in VSMCs^53^. Therefore, we measured the expression level of these factors after manipulating the expression of Lrrc75a-as1. Interestingly, Runx2, Msx2, and Bmp2 expression levels were reduced in VSMCs after overexpression of Lrrc75a-as1, suggesting that Lrrc75a-as1 negatively modulates vascular calcification by decreasing the expression of osteoblast-related factors.

In summary, we showed that Lrrc75a-as1 functions as a negative regulator of vascular calcification, suppressing calcium accumulation and decreasing the expression of osteoblast-related factors. These observations shed light on the possibility that lncRNAs may serve as useful therapeutic targets to suppress vascular calcification and provide insights into the mechanism underlying the role of lncRNAs in vascular calcification.

## Methods

### Cell culture

Primary culture of rat VSMCs was performed as described in our previous study^21^. The A10 cell line derived from the aorta of embryonic rat was purchased from American Type Culture Collection (CRL-1476)^54^ and cultured in Dulbecco’s modified Eagle’s medium (DMEM) supplemented with 10% fetal bovine serum (WelGene). All cells were maintained at 37 °C in a humidified atmosphere containing 5% CO_2_.

### Induction of vascular calcification *in vitro* and measurement of calcium deposition

To induce calcification, the growth medium was replaced with calcification medium [2 mM Pi (pH 7.4) in DMEM medium], and rat VSMCs and A10 cells were maintained in this medium for up to 6 days. The calcification phenotypes including calcium deposition were confirmed in our previous study^21^. We replaced the medium every 2 days. The first day of culture in calcification medium was set as day 0 and calcium deposition was measured at day 3 and day 6.

To determine calcium deposition, the cells were rinsed with phosphate-buffered saline (PBS), and fixed in 0.6 N hydrogen chloride solution at 4 °C for 24 h. The amount of calcium in the supernatant was measured by colorimetric analysis using a Calcium Assay Kit (QuantiChrom™ Calcium Assay Kit, BioAssay Systems) according to the manufacturer’s instructions. Briefly, 7 μL of sample was transferred into a 96-well plate. Working reagent (200 μL) was added to the samples. The absorbance was measured at 570 nm using the Epoch microplate spectrophotometer (BioTek Instruments). Following this, cells were solubilized in 0.1 N NaOH/0.1% sodium dodecyl sulfate to normalize the calcium level. Total cellular protein levels were measured by the Bradford protein assay and used for normalization. Finally, the normalized value of untreated sample was subtracted from that of inorganic phosphate (Pi)-treated sample to calculate the change in calcium deposition.

### Sample preparation for RNA sequencing library construction

Exponentially growing rat VSMCs were divided into four culture dishes containing normal or calcification medium. For the calcification samples, cells were grown in calcification media for 6 h, 3 days, or 6 days (Fig. 1A). At day 6, total RNA was isolated from the cells using TRIzol reagent (Thermo Fisher Scientific). Residual DNA in the samples was removed using DNase I (Takara). For RNA sequencing library preparation, Ribo-Zero Gold rRNA Removal Kit (Illumina) was used, and TruSeq Stranded Total RNA Kit (Illumina) was used for library construction. The library was sequenced using the HiSeq 2500 system (Illumina) in the paired-end mode with 100 sequencing cycles. We performed RNA sequencing in duplicate for each sample.

### Analysis of RNA sequencing data

The process to quantify lncRNA expression is shown in Fig. 1B. To build a reference annotation, we downloaded the annotation files from RefSeq and Ensembl databases^24,25^. These annotations were merged using the Cuffmerge algorithm to obtain the reference annotation for further analysis^55^.

After obtaining the reference annotation, we removed the entire or part of the sequence with low quality reads using Trimmomatic algorithm^56^. After the alignment of filtered reads into the rat genome (rn6) using STAR aligner^57^, the reads were assembled by Cufflinks using our reference annotation as the guide of assembly^55^. The annotations made from each sequencing data were assembled by Cuffmerge, and fragments per kilobase of transcript per million mapped reads (FPKM) was measured using Cuffnorm algorithm based on the newly assembled annotation^55^. We averaged the FPKM from duplicate samples. We only selected those lncRNAs with an average FPKM value greater than 10 and not 0 in any sample. In the criterion to choose candidate lncRNAs based on expression change, we first selected the lncRNAs whose expression was changed more than two fold in any Pi-treated sample compared to that in untreated samples. Among the selected lncRNAs, we only chose the lncRNAs whose expression changed in all three Pi-treated samples or either increased or decreased gradually over time.

### Quantitative reverse transcription polymerase chain reaction (qRT-PCR)

Complementary DNA (cDNA) was synthesized from total RNA using RevertAid Reverse Transcriptase (Thermo Fisher Scientific) and random hexamer primers (Thermo Fisher Scientific). qRT-PCR was conducted using Power SYBR Green PCR Master Mix (Thermo Fisher Scientific) in a Rotor-Gene Q (Qiagen). The primer sequences used for PCR are listed in Supplementary Table 3. Experiments were performed in triplicate. The expression level of each target transcript was normalized to that of glyceraldehyde 3-phosphate dehydrogenase (Gapdh).

### Analysis of the transcript sequences and structures of lncRNAs

To obtain information about the 5′ and 3′ ends of lncRNAs, 5′ and 3′ rapid amplification of cDNA ends (RACE) was carried out with the GeneRacer kit (Thermo Fisher Scientific) using total RNA isolated from rat VSMCs. The gene-specific primers used in this analysis are listed in Supplementary Table 4. The resultant cDNAs were sequenced, which provided information about the 5′ and 3′ ends of lncRNA transcripts. Using this information, we amplified full-length transcripts for each lncRNA, and obtained the exon-intron structures by sequencing the amplicon. The full-length sequences of lncRNAs are shown in Supplementary Table 2.

### Subcellular fractionation

The cells in a culture dish were rinsed with ice-cold PBS, collected by a scrapper, and centrifuged at 6,000 rpm for 5 min at 4 °C to remove the supernatant. The cell pellet was lysed with buffer A (10 mM HEPES, 10 mM KCl, 1 mM DTT, and 0.1 mM EDTA, pH 7.9) and incubated for 25 min on ice. The lysates were treated with 10% Nonidet P-40 and additionally incubated for 2 min on ice. The nuclei were pelleted by centrifugation at 5,000 rpm for 3 min at 4 °C. The remaining supernatant was kept as the cytoplasmic fraction. The nuclear pellet was washed with buffer D (20 mM Tris, 100 mM KCl, and 0.2 mM EDTA, pH 8.0) and centrifuged at 10,000 rpm for 3 min at 4 °C to remove the supernatant. The pellet was lysed with TRIzol LS reagent (Thermo Fisher Scientific) for nuclear RNA extraction. The cytoplasmic fraction from the earlier step was centrifuged at 13,200 rpm for 15 min at 4 °C. The supernatant was collected and treated with TRIzol LS for cytoplasmic RNA extraction. Nuclear RNA was verified by the measurement of pre-mRNA form of Gapdh (pre-Gapdh), and cytoplasmic RNA was verified by the measurement of mature Gapdh and beta-actin (Actb).

### Transfection of plasmid DNAs and small interfering RNAs (siRNAs)

Full-length sequences of lncRNAs were obtained from the cDNA of rat VSMCs using Phusion DNA Polymerase (Thermo Fisher Scientific) and primers (Supplementary Table 5). The PCR products were cloned into pcDNA3 vector and the sequences were confirmed by Sanger sequencing to produce lncRNA overexpression vectors. The plasmid constructs were transfected into A10 cells using Lipofectamine 2000 (Thermo Fisher Scientific) according to the manufacturer’s instructions. For the RNA interference experiment, cells were transfected using Lipofectamine 2000 with 30 nM siRNA (Bioneers) targeting Lrrc75a-as1 (Lrrc75a-as1 siRNA1, sense: 5′-ACG UUG ACA UCG UGG AAU UUU-3′, antisense: 5′-UAU UCC ACG AUG UCA ACG UUU-3′; Lrrc75a-as1 siRNA2, sense: 5′-GGG AAG UUA GGG UGG AAG UUU-3′, antisense: 5′-UCU UCC ACC CUA ACU UCC CUU-3′).

### Alizarin red S staining and quantification

For Alizarin red S staining, cells grown on 24-well plate were rinsed with PBS solution and fixed with 10% formalin for 30 min at room temperature. After three times of washing with distilled water, the cells were stained with 40 mM Alizarin red S solution (pH 4.2, Sigma-Aldrich) for overnight at room temperature, rinsed with PBS, and then photographed by microscopy (Carl Zeiss). To quantify Alizarin red S-stained mineralization, the stain was dissolved with 300 μL of 10% cetylpyridinium chloride (Sigma-Aldrich) for 30 min at room temperature, and 200 μL of extracted solution was used to measure the absorbance at 562 nm using microplate spectrophotometer (BioTeK). The concentration of mineralization was calculated based on the standard graph depicted using the absorbance of standard samples.

### Statistical analysis

If not mentioned otherwise, the statistical analyses were performed by a two-sided paired *t*-test.

## Supplementary information

Supplementary text

Supplementary Table S1
