# Supplementary material for: Long noncoding RNAs in vascular smooth muscle cells regulate vascular calcification
Source: Sci Rep. 2019 Apr 10;9:5848. doi: 10.1038/s41598-019-42283-x (PMC6458154; doi:10.1038/s41598-019-42283-x)
Supplement: Supplementary file 1 — Supplementary text [file 41598_2019_42283_MOESM1_ESM.pdf]

Supplementary information

Long noncoding RNAs in vascular smooth muscle cells regulate vascular calcification

Geon Jeong[1,2,3], Duk-Hwa Kwon[1,4], Sera Shin[1,4], Nakwon Choe[1,4], Juhee Ryu[1,2,3,4], Yeong-Hwan Lim[1,2,3], Jaetaek Kim[1,5], Woo Jin Park[1,6], Hyun Kook[1,3,4,]*, Young-Kook Kim[1,2,3,]*

Supplementary Table S1. A list of lncRNAs identified in this study, along with their expression change after induction of vascular calcification in VSMCs. Those lncRNAs with an average FPKM value greater than 10 and not 0 in any sample are shown. The lncRNAs presented in Figure 2A are highlighted.

Supplementary Table S2. Full-length sequences and the exon-intron boundary of lncRNAs. The exon-exon junction is indicated with asterisk (*).

(A) Linc00116

TGGACTGGAGGAAGCGGAGGCTGCAGGACAAGCTGGCGACGACTCAGAAAAAGCTGGA
CCTGGCCTGAGCACGTTGGGTTCTCTCCAAGCCTCGTGCAGCCCGTATCCGGGGAGCTGC
GCGACTACACGCCCTGAGTCCCGGCTCGTCCTTGCACGGCTTGCAGGAACGTGGCTTGCT
TCCAGACCTCAGAAAGAAAATAGTTTTGTCTTCGCTAACAGCCTGTGCTCAG*CTTGTCG
AAGATGGATATAAAGCACGGGAAAGAAAACATTTCTTGCACTTTATGAATCTATTTTTAA
AATAAAAAATTTTAAACATC

(B) Lrrc75a-as1

CTCTGCGGCCATCATGGGATCATGTTCCTCATCTTCCTGTAGCTGGTACTTTCGCCATGGCC
TGCACTCGGCGGGCCGCAGCCATGCTGAGCCCGGGCCCGGTCTCCCAGCACCGCCCTGGG
CGCTGCGCAGATCTAGGGGAGCCTG*ACAGGACGTTGACATCGTGGAATAGGAGCACTAT
CCCACCATGAG*GTTGGGAAGTTAGGGTGGAAGATTGCCAGCAGCCATGGCCTCCCATGA
G*GGTGTCCCTTTCAAGTTTGAGAACGCACTGGGGGACTGCAGATGGTGTTGACAGCAGC
TGAG*GCTACATTCCCAGGAGTCTCCAGCCATCACCCCAAAAGCAAGGTGTGGGTCAGCC
AACTTTTGTTCTGGACAGGCACGGTTGGTGGTCCTATCTCCCACAGGATCTTCTGTACCAT
TTAATAAAGTTTTGTTTATAAGACTGAT

(C) Snhg1

TCATTTTCCTTTGCTCTGG*GTTTAAGGTACTCTTTTACATGCGAAAGAATGGGAGCACGC
TCTCATTTCAAA*ATATGGCTAATGTCAAAGCCGTTTGCACTTTGGAGCCAGGCCTGTTCA
ATTTTACAAATAGCAAGTTTTTCTTACAATCAG*GGTACGGTGGCAACGTGACTTGAAAC
CCCCAGAATCATACCCGGGATGAATGCAG*GTTTTCTTGGTGGTGATTATCTTAAAG*GA
GGAGGTTGTTCCTGCACGCCATCCATCTCCAGCGTTCGAAG*GTTCTGTTAGCAGTGCTGT
TACTTGGTGCTTCAGAG*ATATGGTGACTGGCCGAGCGCAAAGGAGCCATGCCAG*GAAA
ACTAAGAGCACATCTTCATAAAGTCCAGGCTAAAGTAAG*GTCTGAGGAGAGGGATGAG
GTGTGTGTTAGAG*GAACTGAAATCAAAGATCCGATTACCTAGGCCAGTGGCACGGCAA

ACTTCAGAACCCATGGAATACAGCCTGTTGATTCCTTGTGGATTTTTAGCATCTGAGCAG
TATCAGTGGCACTCCAAGTGAAATGGTTGATCTCTTCAGTACCGCTGGAGCAGATTAATA
TTTATTTCACTGCCAGAGGCTATATTTCTCTGCCCCCCCATACCCCCTTCCTCTTCTCCTTA
GACTACTTAAGATTGAAATATTTGCAACTCCAAATTTATCTTCCTTGCTTACCACTTTTTT
TTCGGAGCTGGGGACCAAACCCAGGGCCTTGCGCTTCCTAGGTAAGCGCTCTACCACTGA
GCTAAATCCCCAGCCCCGCTTACCACATTGTTAATCTGATCCTTCCGCAGTTATTTGTGCT
GTTCTTTTTGTTCCTGGCAATAAATGCTTTTTATGCTA

(D) Snhg16

CTTTCCGAGGTCCTCTGCCGCGTGCACTAAAGTTATGGCCGGTCCTGTCCGTGGTTCACTC
TCTGAAAGGCCTAGGGTTTGGGTGATG*GCACGCCATTGCACATGGGACGCAAGCCGCA
GGTTCCATTTCAGAAGTAATGGCTGGATCCCTGGAAGCAGATGGACTCGGTGTGGTTAGA
GGGCAGAGGTAGATGATGCTGCGTATTGAGCCCTCCTCAGCCCACTTTG*ATTGAGGTGT
TGCGCGGTGAGGAAGGGCCTTCGTGACCAGTCTGCATG*GGGGAGGCTGAACCATAGTTT
CTGGGCCTTCAAGGGGTGTTGCTGCAGCCACGGGGGCAGCCCTCCTGCGCCTGGACTTCT
GCCTGCGAGCAGAGGAGCTTCCACACACAGCTCGTCAGAGCATCCGTGCTTCTGCGCTAA
GTGGCAG*ATTCCAGGACGGCTCCAGGGGGCATCATCGAAAGGCGTGGCACATCAGGGC
CTTCAGGATGGCTGCGTCCTG*GTTATTGCCCTGTGGTGCCAGAGAGAACCACACAGCCT
GCTGCTGCTCCTGCCTTTAATTTCCCAAAGAGCCATCAAAAGACCTTGGACTTTGTTCTGC
AAGAGGAGCTAGCAAAATGGAGTGACTGTGGTCACCCTGGAAGCCTTTGACAGCTGGGG
AGGCCCTGCTGAGCTTAGAAAACCATCTGATAGATTTCCTGAGTAAGGATTGGGCTTTGT
ATTTTGAAGCACAAATAAACTTGATAAAATTAAA

Supplementary Table S3. The sequences of PCR primers to measure lncRNA and mRNA expression.

Linc00116 forward primer	5′-TTGCTTCCAGACCTCAGAAAG-3′
Linc00116 reverse primer	5′-TCCCGTGCTTTATATCCATCTTC-3′
Lrrc75a-as1 forward primer	5′-TTTCGCCATGGCCTGCACTC-3′
Lrrc75a-as1 reverse primer	5′-GCCATGGCTGCTGGCAATCT-3′
Snhg1 forward primer	5′-CAAAGATCCGATTACCTAGGCC-3′
Snhg1 reverse primer	5′-CACAAGGAATCAACAGGCTG-3′
Snhg16 forward primer	5′-TCGAAAGGCGTGGCACATCA-3′
Snhg16 reverse primer	5′-CTCTGGCACCACAGGGCAAT-3′
Malat1 forward primer	5′-AGTTCTGTTGGCACACTGGGT-3′
Malat1 reverse primer	5′-CCAAAGCTGCACTGTGCTGTA-3′
Gas5 forward primer	5′-ACATGTGGGTCCCTTTAACTG-3′
Gas5 reverse primer	5′-GGCAAATCTTCTGTTCAAGCATC-3′
Runx2 forward primer	5′-CGATGGGACCGTGGTTACCG-3′
Runx2 reverse primer	5′-GTCCGCTCCGGCCTACAAAT-3′
Msx2 forward primer	5′-GGCCTCTCGTCAAGCCCTTC-3′
Msx2 reverse primer	5′-AGTATCTGCCGGGCTCCTGT-3′
Bmp2 forward primer	5′-CGACGTCCTCAGCGAGTTTG-3′
Bmp2 reverse primer	5′-GGGTGGGTCTCTGCTTCAGG-3′
Pre-Gapdh forward primer	5′-CCATGGTGCAGCGATGCTTT-3′
Pre-Gapdh reverse primer	5′-ACGGCCAAATCTGAGGCAAG-3′
Gapdh forward primer	5′-ATGACTCTACCCACGGCAAG-3′
Gapdh reverse primer	5′-CTGGAAGATGGTGATGGGTT-3′
Actb forward primer	5′-CACTTTCTACAATGAGCTGCG-3′
Actb reverse primer	5′-CTGGATGGCTACGTACATGG-3′

Supplementary Table S4. The sequences of gene-specific primers (GSPs) used in RACE experiments.

Linc00116 5′ GSP	5′-GAGCACAGGCTGTTAGCGAAGAC-3′
Linc00116 Nested 5′ GSP	5′-AAGAGCAAGCCACGTTCCTGCAAG-3′
Lrrc75a-as1 5′ GSP	5′-CTATTCCACGATGTCAACGTCCTG -3′
Lrrc75a-as1 Nested 5′ GSP	5′-AGGCTCCCCTAGATCTGCGCAG-3′
Snhg1 5′ GSP	5′-TTCAAGTCACGTTGCCACCGTACC-3′
Snhg1 Nested 5′ GSP	5′-GTGCAAACGGCTTTGACATTAGCC-3′
Snhg16 5′ GSP	5′-CCAGGGATCCAGCCATTACTTCTG-3′
Snhg16 Nested 5′ GSP	5′-GCTTGCGTCCCATGTGCAATGG-3′
Linc00116 3′ GSP	5′-GGCTTGCTTCCAGACCTCAGAAAG-3′
Linc00116 Nested 3′ GSP	5′-TCTTCGCTAACAGCCTGTGCTCAG-3′
Lrrc75a-as1 3′ GSP	5′-ATTCCCAGGAGTCTCCAGCCATC-3′
Lrrc75a-as1 Nested 3′ GSP	5′-AAAGCAAGGTGTGGGTCAGCCAAC-3′
Snhg1 3′ GSP	5′-TCTGAGGAGAGGGATGAGGTGTG-3′
Snhg1 Nested 3′ GSP	5′-CAAAGATCCGATTACCTAGGCCAG-3′
Snhg16 3′ GSP	5′-TGGAGTGACTGTGGTCACCCTGG-3′
Snhg16 Nested 3′ GSP	5′-ACCCTGGAAGCCTTTGACAGCTG-3′

Supplementary Table S5. The sequences of PCR primers for cloning of lncRNAs.

Linc00116 forward primer	5′-TGGACTGGAGGAAGCGGAGGCTGC-3′
Linc00116 reverse primer	5′-GATGTTTAAAATTTTTTATTTTA-3′
Lrrc75a-as1 forward primer	5′-CTCTGCGGCCATCATGGGATCAT-3′
Lrrc75a-as1 reverse primer	5′-ATCAGTCTTATAAACAAAACTTT-3′
Snhg1 forward primer	5′-TCATTTTCCTTTGCTCTGGGTTT-3′
Snhg1 reverse primer	5′-TAGCATAAAAAGCATTTATTGCC-3′
Snhg16 forward primer	5′-CTTTCCGAGGTCCTCTGCCGC-3′
Snhg16 reverse primer	5′-TTTAATTTTATCAAGTTTATTTG-3′

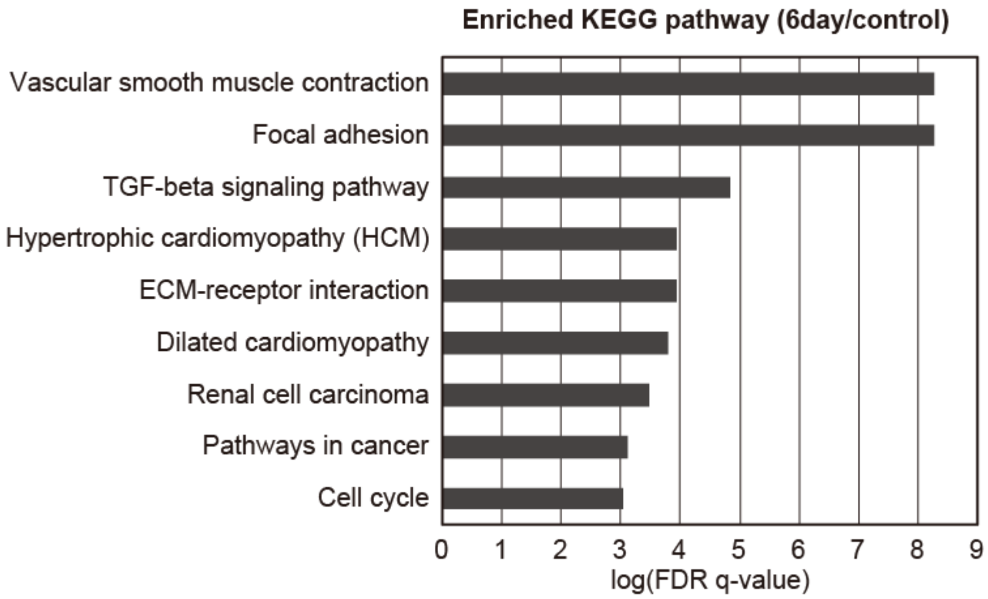

Supplementary Figure S1. Pathway analysis of RNA-seq data. The decreased mRNAs at the day 6 sample compared to the control sample with fold change (6 day/control) below 0.5 were selected. Pathway analysis was performed using the Kyoto Encyclopedia of Genes and Genomes (KEGG) gene set analysis (reference) at MSigDB database [22,23].

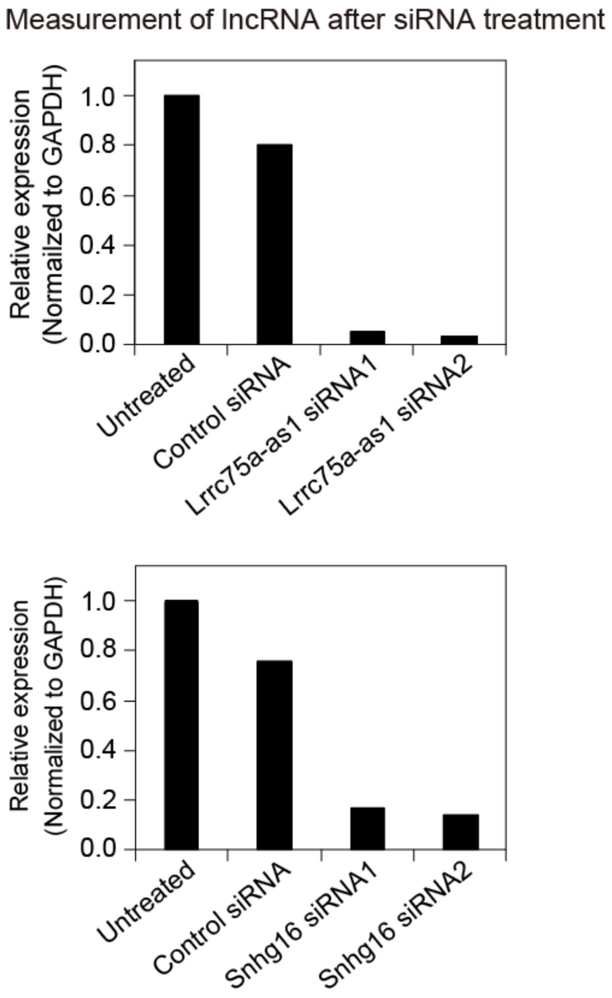

Supplementary Figure S2. Measurement of lncRNA level after the treatment with siRNA for each gene.

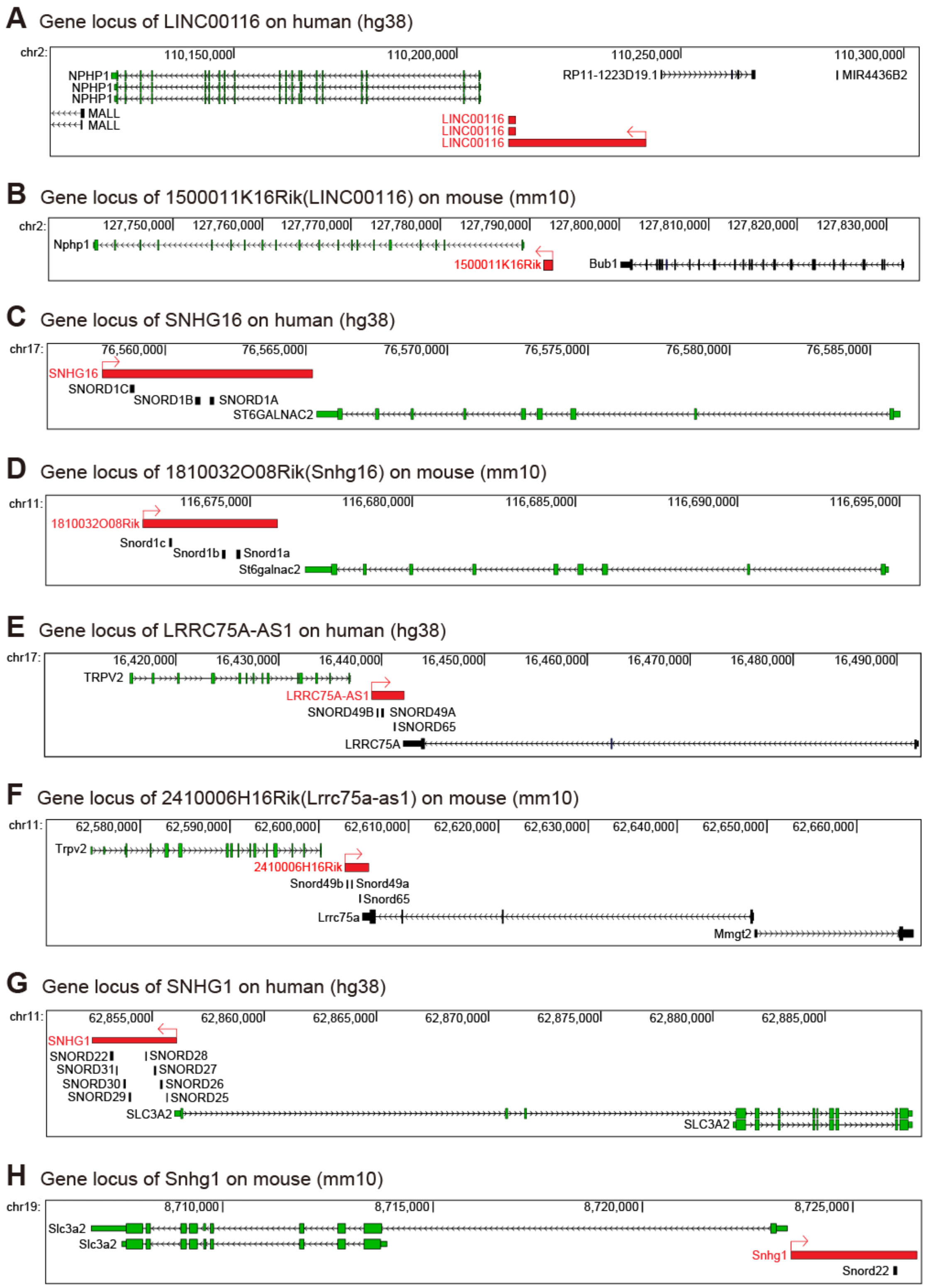

Supplementary Figure S3. The human and mouse genomic regions near lncRNAs analyzed in this study.

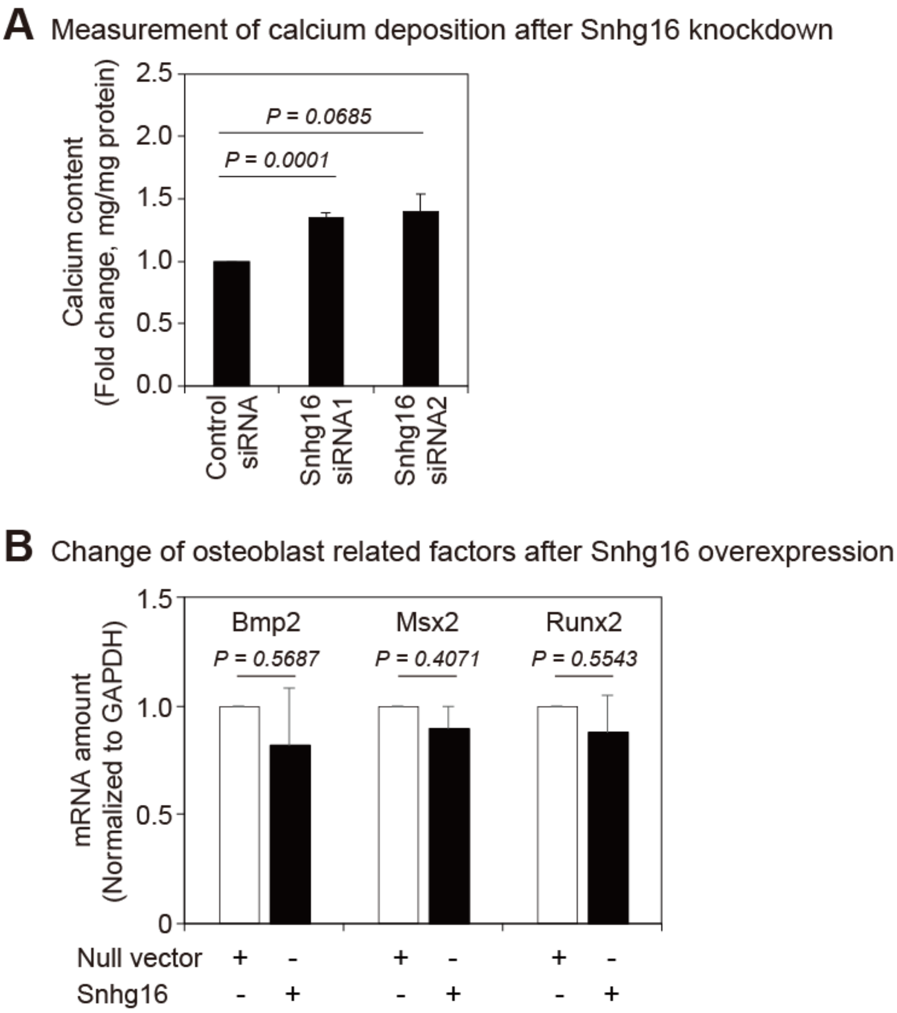

Supplementary Figure S4. (a) Measurement of calcium deposition after knockdown of Snhg16. Six independent experiments were performed, and the P value was calculated by a two-sided paired *t*-test. (b) Expression change of the osteoblast-related factors after overexpression of Snhg16. Error bars indicate standard error between three independent experiments.

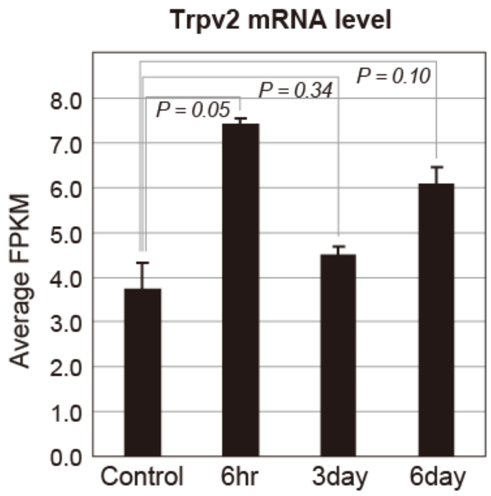

Supplementary Figure S5. The expression level of Trpv2, the neighboring gene of Lrrc75a-as1. FPKM values from RNA-seq data were averaged. Error bars indicate the deviation from two samples.
